# 

**Published:** 1891-08-15

**Authors:** 


### NOTICE TO CORRESPONDENTS.

All MS., Letters, Books for Review, and other matters intended for the Editor should be addressed The Editor, The Lodge, Porchester Square, London, W.

The Editor cannot undertake to return rejected M.S., even when accompanied by stamped directed envelope.

**Notice to Advertisers and Subscribers.**

All Advertisements, Orders for Copies of The Hospital, and Business Communications should be addressed to The Publisher, *not to the Editor*, The Hospital, 140, Strand, W.C.

The Cost of Subscription, post free, is as follows:—Per week, 1½d.; per quarter, 1s. 8d.; per half-year, 3s. 3d.; per annum, 6s. 6d. Foreign:—Per annum, 8s. 8d.; payable, in all cases, in advance.

**NOTICE.—The Index for Vol. IX., (ending March, 1891) is on sale at the Office of this Journal, price 2½d., post free.**

### General Charities.

[This Column is devoted to an account of work of charitable institutions other than Medical Charities. The Editor will be glad to receive reports or news of work at 140, Strand. *Philanthropy* should be plainly written on the envelope.]

We have received an appeal from **THE LADIES' CHARITY SCHOOL**, Powis Gardens, Notting Hill, W. The school receives from any part of the United Kingdom and trains for domestic service the daughters of respectable parents in necessitous circumstances. The children, who are received between the ages of eight and ten, are educated, clothed, and maintained until the age of fifteen. It being considered that technical education will be advantageous, the committee propose to allow girls likely to be benefited thereby to remain till the age of sixteen, provided their friends are willing to contribute towards the extra expense of lessons in cooking, dressmaking, cutting-out and getting-up fine linen. Cooking surely should be part of the training of all the girls in the school.

Institutions which, from the nature of their work, are unable to make a great display of the fruits of their work, or of the misery which they endeavour with more or less success to relieve, find it far from easy to gain the support required in order to carry on their operations efficiently. This difficulty is one with which the **ZENANA MISSIONS** have to contend. Not only are they unable to show the result of their work to those to whom they appeal in this country, but rival claims nearer home have the first call. Not only is support constantly required for the present work of the missions, but increase of support is needed for the ever-recurring necessary extension of the work. Owing to scarcity of helpers the work in Bombay during the past year has been carried on under considerable difficulties, and at least two more missionaries are now required; what applies to Bombay applies with equal force to other large centres in India.

During the past summer, we use the word with some hesitation, as we are in doubt as to whether there are more than the three seasons of spring, autumn, and winter in this country, a new wing has been added to **THE SCHOOL OF HANDICRAFTS FOR DESTITUTE BOYS**, at Chertsey. This wing has been erected at a cost of £1,200, the gift of an anonymous benefactor, but though the actual building was done by a builder, the whole of the interior fittings, in addition to the woodwork, painting, and glazing, were done by boys trained at the school. For some time there had been a want of dormitory accommodation, and it has not been possible to provide room for more than seventy boys. The new building has remedied this state of affairs, which is most satisfactory, seeing that the addition of fifty or more boys is found to add little to the current expenses beyond the cost of food and clothing, the working staff of officers being quite sufficient to cope with many more boys than the institution could hold. The committee, with a view to perfecting the work of the institution, are anxious that Parliament should come to their assistance with enactments, that whenever a neglected child is found without parent or guardian able and willing to nurture and train it until at a suitable age it is competent for self-support, that it be placed in an industrial school able and willing to fulfil this duty; and that whenever a child has been thus transferred, the natural guardians lose their civil rights over the child, and be compelled to pay whatever it may be proved is possible, in support of the expenses to the school undertaking their duties.

A visit to the Royal Naval Exhibition will have brought before the minds of many who have seen the full-sized model of the "Victory" stories of the "seamanship" of our forefathers. That seamanship which was the pride of every Englishman is no doubt, as Mr. Forwood pointed out during his inspection of the **TRAINING SHIP "ARETHUSA,"** practically a lost art. This is due chiefly to the introduction of machinery on such a large scale in our modern ships of war. One branch of the numerous duties performed on the "Arethusa" which receives encouragement worthy of it is seamanship, so that it is to be hoped the art will not be entirely lost. The "Arethusa" is connected with the National Refuges for Homeless and Destitute Children, the work of which is now so well-known as hardly to need a reference to its beneficent character—a beneficence which gives to the thousands of children in its different institutions the most useful of all educations: a technical one, which fits them to help themselves when of an age to enter into the world. It appears that very few boys trained on the "Arethusa" enter the British Navy, owing to the fact that the parents regard their children in the light of money-making machines (we do not mean the automatic instrument which presents you with a sweet in exchange for a penny), and prefer to find them more lucrative employment. Mr. Forwood is of opinion that the parents are herein short-sighted and too oblivious of the opportunities for intelligent, sharp, sober, and industrious boys getting promotion in the Navy. He evidently expresses the views of the Admiralty on this point, who are desirous of bringing the training-ships generally into closer connection with the Royal Navy. With a view to this it is the intention of the Admiralty to offer a gunnery instructor to the ship, who will be a pensioner from the Royal Navy. Those who pass can be entered into the Royal Naval Reserve, and earn a capitation grant for the ship, and the ship will receive a grant of £25 for every first-class boy. These training ships should be an excellent recruiting ground for the Navy.

### Scraps and Gleanings.

It has been decided to extend the accommodation at the Luton Cottage Hospital.

The influenza epidemic has reappeared in Moscow, and as many as 500 persons daily have been attacked by the malady.

A successful demonstration in connection with the Hospital Sunday Fund was held at Whittington Moor on Sunday last.

At the annual meeting of the subscribers of the Monkwearmouth and Southwick Hospital it was decided to add a female ward to the institution.

The Southampton Hospital Sunday Fund has made steady progress. The collection, which amounted to £548 in 1873, has increased to £1,083 in 1891.

As a result of the recent demonstration in North Kensington, Paddington, and Marylebone, a sum of £40 has been handed over to St. Mary's Hospital.

The action of the Great Northern Central Hospital in refusing treatment to a working man in receipt of thirty shillings a-week, has given rise to a great deal of discussion.

The *Gentlewoman* has lately had a number of competitions for children. Among the prizes offered was one for the best plum-cake; and the cakes sent in were afterwards distributed among the hospitals.

We regret to learn from the annual report of the Central London Ophthalmic Hospital that half the wards are still closed for want of means. We trust the charitable public will soon remove this limitation of a useful work.

The annual report of the Liverpool Northern Hospital shows that the work has been carried on in a very satisfactory manner. The Horse Ambulance in connection with the institution has proved of the utmost benefit to the public.

At the annual general meeting of the Governors of the Lewes Dispensary and Infirmary and Victoria Hospital it was decided to adopt the system of free tickets, and to admit of this subscribers were asked to increase their subscriptions.

The hopes hitherto entertained that famine would be averted in the districts of Chingleput and North Arcot, India, have now been almost abandoned. The heat there is unprecedented, and the rains have been insufficient to save the crops.

The annual Saturday house-to-house collection in aid of the funds of the Grantham Hospital took place on Saturday last, when nearly every house in the borough received a visit from one of the collectors appointed, and the sum of £58 was collected.

A very successful result has attended the Norwich Hospital Saturday collection. The proceeds amounted to £228. Such was the general desire of all to assist, that an organ grinder devoted himself to the cause, and collected the sum of eleven shillings in its behalf.

The Suffolk Convalescent Home, Felixstowe, has extended its sphere of usefulness during the past year by additions to the women's wing, effected mainly through the generosity of Mr. W. Cuthbert Quilter, M.P. The institution is intended chiefly for patients requiring the benefit of sea-bathing.

The Earl of Coventry (Lord-Lieutenant of Worcestershire) opened a new Cottage Hospital at Bromsgrove, in the presence of a large company, on the 4th ult. The hospital, which is built upon the pavilion system, has been erected from the designs of Messrs. Cotton and Bidlake, architects, Birmingham.

On Friday evening, at the Guild Hall, Carnarvon, the annual meeting of the Cottage Hospital was held. The report showed that the subscriptions have fallen off, and that the institution is now indebted to the extent of £24 17s. 5d. to the bank, and this in spite of every effort to keep down the expenditure.

The Ladies' Committee of the Ancoats Hospital have set an example of what can be done by combined energy and enthusiasm. Only a month ago a scheme for the establishment of a convalescent home was suggested by a member of the committee, and now, by means of personal labours and appeals to the public, a small home is already established in the village of Wilmslow.

The annual general meeting of the Governors of the Hereford Dispensary was held on the 30th ult. The Committee reported that the work carried on by the institution during the past year had been satisfactory, although the patients who brought letters of recommendation from subscribers and those relieved on application were fewer than last year, the numbers being 3,640, as compared with 4,171 last year.

At the 101st half-yearly general meeting the governors of the Metropolitan Convalescent Institution, it was reported that 2,593 poor convalescent patients had been received into the three homes during the past six months. The contributions to the general fund were: Annual subscriptions, £2,275; donations, £680. The contributions to the Seaside branch were: Annual subscriptions, £1,391; donations, £372.

At the annual meeting of the subscribers to the Retford Dispensary and Cottage Hospital an additional rule of some interest was made. It provided that, "In all cases where a patient has to undergo an operation, the medical man who has been accustomed to attend the patient before admission may be present during the operation by the invitation of the medical officer operating, or at the request of the patient."

The two youngest defendants ever summoned before the Southampton County Bench appeared before them recently. The youngest was a baby two and a half years old, who had to be carried in its mother's arms. The co-defendant, a brother, aged five years, was so small that he had to stand on the dock seat to be seen. The charge against them was one of damaging peas, but the magistrates peremptorily refused to hear the case.

The Hinckley Cottage and Nursing Institute was opened on the 30th ult. by Lady Agnes Tollemache Scott. Mr. H. Atkins, the Hon. Secretary, announced that the establishment of a cottage hospital in Hinckley was chiefly owing to Dr. Pritchard. His appeal for funds was so generously responded to that it was thought well to combine with the district nursing plan a cottage hospital, where a Matron could preside, and beds be provided for the sick that could not be properly nursed in their own homes.

### THE STUDENT OF MEDICINE.

[All communications for this Supplement should be addressed to The Editor of The Hospital, at 140, Strand, with the words, "Students' Column," written in the left hand top corner of the envelope.]

### ON GERMAN STUDENTS (concluded).

*The substance of a paper read before the St. Mary's Hospital Medical Society by* W. Overend, *B.A. Oxon.*

I will now describe the examination in midwifery. In this branch every student must examine and deliver in the presence of an assistant. A review and criticism of the case must be written during the same day, and the patient must be visited for a week, and notes of the case written up. A vivâ voce on a dummy and diagnosis of presentations, &c., are also required. In ophthalmology, a somewhat similar course is pursued. Several cases must be described and diagnosed, to one of which special attention must be paid and visited for several days. The subjects of anatomy and physiology must be taken again in the final, and the latter includes practical dissection, a paper of questions, and osteology. In physiology they must criticise and discuss one or two physiological questions. The main feature of the examination is the few paper questions and the large amount of oral examination. Having passed the final examination successfully, a qualified man may take his M.D. or not, as he chooses, there being no intermediate M.B. examination. Should he wish to do so, although the conditions vary somewhat in the different universities, practically there is nothing more for him to do than to write a thesis on some medical subject, under the personal supervision of the professor in that branch. The student generally spends a month in getting up the literature of the subject as recommended by the professor, the thesis itself requiring four or five months' close observation and application. This must be approved of by three professors, after which he receives his diploma, and his thesis is printed and a copy sent to every university throughout Germany. In comparing the two methods of teaching, we must not shut our eyes to the fact that the German character is essentially different from our own. We have the reputation throughout the Continent of being essentially practical men, and an exact imitation of their methods in a large centre of education like London would be impracticable and impossible. Moreover, the relation of the State to their medical institutions is absolutely different from our own. Nevertheless, we can learn something from them. We must at all risks keep our own system intact with regard to its practical aspect, that is, the frequent attendance of our students in the wards and the post-mortem-room and our clerkships and dresserships. We might increase our clinical lectures, so as to make three weekly, in surgery and medicine, and conduct them on a somewhat similar pattern, since they are practical, with actual demonstrations and examinations. This would increase the fluency of our students and there would be no danger of this passing into illogical verbosity. We think the special subjects, as the eye, ear, throat, including bacteriology, might be made a little less optional. From what has been said it follows that the German schools are exceedingly useful to our newly qualified men. A year may be profitably spent in Germany, provided the knowledge of the German language is sufficiently extensive and accurate. In this way the gap as regards the scientific aspect would be filled up. In the first lecture given by Czerny he touched upon the question of German medical education and attempted to combat the ideas of those German professors who asserted that their teaching was too theoretical, too scientific, and not sufficiently practical. Czerny necessarily took a one-sided view of the question, being not sufficiently acquainted with our own methods. If one wishes to study experimental pathology or physiology the universities of Strasburg, with its galaxy of renowned teachers, splendidly equipped laboratories, or Leipsig or Heidelberg with Czerny, Erb, Kuhne, Gegenbauer and Arnold, should be selected. On the other hand if one wishes to study clinical pathology, Berlin, Vienna, or Paris are more suitable. But it is useless unless one understands the language, and for this three or four months of hard work at least are requisite. These must be spent in some remote spot where few English can gain access. I have only been able to deal with Heidelberg, Tubingen, and Strasburg. My deductions have been drawn from about eighteen months' residence at these universities. They must, therefore, be somewhat limited and liable to correction. Before concluding, however, I should like to pay a tribute of respect to the German professors themselves. Passing rich and contented with an annual salary of £400 or £500, they seem to be absolutely contented with their lot, possessing a sincere and intense enthusiasm for research, and as a general rule with unbiassed minds entirely open to conviction and ever ready to give a helping hand and sound advice to the

### The Hospital Advertiser.

THE STUDENT OF MEDICINE—(*continued*).

foreigner, especially Englishmen, who work under their direction.

### NOTES FROM THE SCHOOLS.

**University of Oxford.**

The Regius Professor of Medicine has started a new department for the study of Bacteriology. Dr. Menge, from the Munich University, has been appointed to take charge of this section. Both students and medical practitioners are attending the course of practical instruction in the various branches of Bacteriology.

**University of Cambridge.**

At Cambridge University, during the past academical year, nineteen M.D. degrees, seventy-two M.B. degrees, and seventy B.C. degrees have been conferred. This shows a very steady annual increase of medical graduates of this school. Mr. Adami, the John Lukas Walker Student in Pathology at Cambridge University, having been elected to a fellowship at Jesus College, has had to resign his studentship. The vacancy has been filled by the election of Mr. A. A. Kanthack, B.A., M.B., B.Sc. Lond., F.R.C.S., who is at present at work at Simla on the Leprosy Commission. The directors also granted Mr. E. H. Hankin, of St. John's College, £60 towards the cost of bacteriological apparatus required by him for his researches.

**Liverpool.**

The chair of physiology in University College, Liverpool, lately endowed by Mr. George Holt with £10,000, has been filled by the appointment of Mr. Francis Gotch, who for the past eight years has been Lecturer on Physiology at Oxford University.

### STUDENTS' MEDICAL SOCIETY.

**Westminster Hospital.**

The Guthrie Society held its annual general meeting on Thursday, July 16th. Mr. Stonham, the President, was in the chair, and he was supported by Messrs. H. A. Stonham, Mills, and Campbell. The Treasurer's report was read and adopted on the motion of Mr. Yearsley. The Secretary then read his annual report, in which it was stated that twenty new members had been admitted since the last annual meeting, and that the total number of attendances for the nine meetings had been 285, making an average of thirty-one for each meeting. The list of the papers read before the Society was also described, and the report was adopted on the motion of Mr. Cullinans. The officers for the ensuing year was the next item dealt with, and the ballot for the office of Vice-President resulted in the election of Mr. P. M. Yearsley, who received five votes against Mr. Gossage, who obtained three. The retiring President then made a few remarks, and congratulated the members upon the flourishing condition of the Society, and the annual meeting terminated with a vote of thanks to the Chairman proposed by Mr. Mills. The following were elected officers for the ensuing year: President, Dr. W. H. Allchin, M.B., F.R.C.P.; Vice-President, Mr. P. Macleod Yearsley, M.R.C.S., L.R.C.P.; Hon. Secretary, Mr. Brice; Hon. Treasurer, Mr. Eric H. Sharman, M.R.C.S., L.R.C.P.; Committee, Messrs. H. V. Morris, H. H. Mills, J. A. L. Campbell, and W. B. Winckworth.

### APPOINTMENTS.

At the University College, London, G. F. Blacker, F.R.C.S., has been appointed senior demonstrator of anatomy. At the Birmingham General Dispensary, C. T. Congreve Barber, M.R.C.S., L.R.C.P., has been appointed resident surgeon. At the Royal Hospital for Sick Children, Edinburgh, J. H. Beilby, M.B., C.M. Edin., and J. A. Robertson, M.B., C.M.Glas, have been appointed resident physicians. At the Hospital for Women, Soho Square, Ernest H. Brock, M.B., B.S., L.R.C.P.Lond., M.R.C S.Eng., has been appointed house physician. At the Bootle Borough Hospital, Thomas L. Gilcriest, L. and L.M.R.C.S.I., L. and L.M.R.C.P.I., has been appointed senior, and W. W. Cleemey, L.R.C.P.Lond., M.R.C.S.Eng., assistant house surgeon. At the Kidderminster Infirmary and Children's Hospital, Fred. J. Green, B.A., M.B., B.Ch., B.A.O.Dub., has been appointed house surgeon. At the Newark Hospital, Ernest Ringrose, M.R.C.S., L.R.C.P., has been appointed resident medical officer.

### VACANCIES.

The following vacancies are announced this week, the amount of salary in each case being given after the name of the institution:

**Resident Medical Officers.**

Coventry and Warwickshire Hospital, House Surgeon—Salary £100, and board, &c.

Derbyshire Royal Infirmary, Resident Assistant House Surgeon—Salary £10 for first six months, £25 for second six months.

Leeds Union, Assistant Medical Officer for the Workhouse Schools—Salary £100, and board, &c.

### The Hospital Advertiser.

### THE STUDENT OF MEDICINE—(continued).

Rotherham Hospital, Resident House Surgeon—Salary £100, with board, &c.

Royal Albert Hospital, Devonport, Assistant House Surgeon—Board, &c.

Stockport Infirmary, House Surgeon—Salary £100, with board, &c.

### SCHOLARSHIPS AND PRIZES.

**London School of Medicine for Women.**

The prizes for the winter session, 1890 91, have been awarded as follows: Physiology, Miss Corthorn and Miss M. Benson (equal); chemistry, Miss Leney; organic chemistry, Miss S. Hughes; anatomy, first year, Miss Appel? anatomy, second year, Miss Boyle; medicine, Miss E. M. Harris; surgery, Miss Pace; operative midwifery, Miss Wheeler. Summer session, 1891: Practical organic chemistry, Miss E. Knight; practical chemistry, Miss Yardley; practical histology, Miss Benson; and materia medica, Miss Moffett.

**Edinburgh School of Medicine for Women.**

Thirteen students presented themselves at the recent professional examinations held in Edinburgh by the conjoint Colleges of Physicians and Surgeons, and twelve of them successfully passed their examinations. Eight were successful in the first professional, viz., Kate Clutterbuck, Kate Cornford, Elizabeth Erskine, Elizabeth Gilchrist, Amy Lillingston, Mary M'Dougal, Margaret Macnaughton, and Isabella Venters. In the second professional, Mabel Blackwood. In the third or final examination, Elizabeth Christie, Alice Moorhead, and Emily Thomson; the three last being now entitled to place their names on the Medical Register.

### PASS LISTS.

**Royal College of Physicians and Surgeons of England.**

The following gentlemen, having passed the necessary examinations, have been admitted diplomates in public health, by the Colleges of Physicians and Surgeons of England, viz.: Messrs. Charles James Acton, L.R.C.P Lond., M.R.C.S.Eng., of University College Hospital; David Stuart Erskine Bain, M.R C.S.Eng., of Charing Cross Hospital; Henry Beale Collins, M.R.C.S.Eng., of King's College Hospital; Charles Fillingham Coxwell, M.B.Camb., M.R C.P.Lond., of St. Thomas's Hospital; Alfred Cropley, M.R.C S.Eng., of London Hospital; William Gilbert Dickinson, L.R.C.P.Lond, M.R C.S.Eng., of St. Thomas's Hospital; Evan Evans, M.R.C.S.Eng., of St. Bartholomew's Hospital; Septimus Farmer, M R.C.S.Eng., L.R C.P.Edinb., of King's College Hospital; Francis Turner Frost, L.R.C.P.Lond., M.R.C.S.Eng., of Guy's Hospital; Alfred John Gregory, M.B.Durh., M.R.C.S.Eng., of London Hospital; John Morrison Hobson, M.D.Edinb., L.R.C.P.Lond., and M.R.C.S.Eng., of Edinburgh University and Guy's Hospital; William More Hope, M.R.C.S.Eng., of University College Hospital; Sorab Cowasji Hormusji, L.R.C.P.Lond., M.R.C.S.Eng., of Bombay Medical College and University College Hospital; Frederick David Irvin, M.B. Lond., M.R.C.S.Eng., of University College Hospital; Alexander Napier Ledingham, M.B.Glasgow, of Glasgow University and University College Hospital; Charles Edward Matthews, M.B.Oxon., L.R.C.P.Lond., and M.R.C.S.Eng., of Oxford University and St. Thomas's Hospital; Charles H. Piesse, M.R.C.S.Eng., of King's College Hospital; George Pires, L.R.C.P.Lond., M.R C.S.Eng., of Bombay Medical College and University College Hospital; St. Clair Shadwell, L.R.C.P.Lond., M.R.C.S.Eng., of Guy's Hospital; Richard Rawdon Stawell, M.D.Melbourne, of Melbourne University; Francis Joseph Stevens, M R.C.S. Eng., of Oxford University and St. Bartholomew's Hospital; Charles Henry Wade, L.R.C.P.Lond., M.R.C.S.Eng., of Oxford University and London Hospital; Philip Henry Whiston, L.R.C.P.Lond., M.R.C.S.Eng, of St. Thomas's Hospital; and William Atkinson Wood, M.D.Melbourne, of Melbourne University.

**University of Oxford**

Second M.B. Examination.—The following have passed: C. P. Lovell, St. John's; A. B. Roxburgh, Exeter; F. H. Burton-Brown, Magdalen; A. M. Possage, Magdalen; G. Jones, Oriel; E. J. Moore, Christ Church; L. E. Parkhurst; Jesus; G. E. Pritchard, Hertford; E. T. Withington, Balliol.

First M.B. Examination.—The following have passed in anatomy and physiology: A. Butler-Harris, Trinty; G. J. Conford, Christ Church; H. Cooper, Queen's; H. R. Hickman, Christ Church; L. L. Jenner, Trinity; W. J. Maurice, Keble; R. J. Porter, Pembroke; F. C. Poynder, St. John's; W. Ramsden, Keble; E. H. Saunders, St. John's; F. W. Walton, Keble.

**University of Edinburgh.**

The following 185 gentlemen received the degrees of M.B. and C.M. on Aug. 1st.:—W. Adam, Dingwall; A. J. Anderson, Portobello; A. W. Anderson, St. Andrews; Jas. Anderson, Edinburgh; John Anderson, Shetland; R. J. Ashton, Lavender Hill; F. R. B. Atkinson, Brighton; Jas. Atkinson, Harrogate; L. G. Barbeau, Edinburgh; J. H. Bayley, Northampton; Hugh Bennett, Edinburgh; R. J. A. Berry, Ormskirk; A. J. Bienemann, Brighton; T. H. Bishop, Gloucester; Hight Blundell, Sevenoaks; J. W. Bone, Newcastle-on-Tyne; G. E. Bowker, Bath; J. J. Brennan, Marchmont-cres.; T. B. Brierley, Cheshire; J. D. Broadfoot, Edinburgh; S. C. Brush, Dundee; W. M. Cairns, Liverpool; K. M. Cameron, Edinburgh; Percy Capper, Liverpool; E. J. W. Carruthers, Inverness; J. W. N. Chapman, Wales; H. A. Clark, Glasgow; G. E. Clemons, Tasmania; Chas. Cochrane, Worcester; R. P. Cockburn, Ealing; C. J. van Coller, Edinburgh; The Rev. J. W. Compton, Hants; John Cowan, New Galloway; Robt. Cran, Fearn; J. H. Crawford, Perthshire; J. F. Crombie, N. Berwick; H. W. Crosse, Norwich; G. D. Darlington, Edinburgh; Jas. Davidson, Edinburgh; H. R. L. Davies, Bewdley; R. G. Dempster, Edinburgh; W. B. Drummond, Edinburgh; Alf. Duke, Brechin; Samuel Edgerley, Portobello;

### The Hospital Advertiser.

### THE STUDENT OF MEDICINE—(*continued*).

George Elder, Haddington; A. C. Elliott, Bradford-on-Avon; Michael Emin, Warrender Park Terrace; F. W. Eurich, Bradford; J. L. Fenton, Ebinburgh; L. G. Fink, Edinburgh; E. C. Fischer, St. Andrews; J. L. Fletcher, Manchester; W. D. Forsyth, Berwickshire; F. T. Foster, Edinburgh; E. J. Fox, Preston, Lancs.; John Francis, Grimsby; H. E. Fraser, Inverness; E. B Fuller, Edinburgh; W. M. D. Gallie, Edinburgh; Denson Gibb, Midlothian; H. J. Glover, Gatehouse; Fred. Gourlay, Dundee; F. C. H. Grenier, Ceylon; Wm. Griffith, Milford Haven; W. C. Grosvenor, Glasgow; H. B. Hall, Bedford; S. A. Harris, Midlothian; S. H. Hartley, Bradford; A. J. Haslam, Westminster; P. T. Hatton, Leamington Spa; Edmund Hay, Edinburgh; W. P. Hay, Arbroath; R. A. Heath, Buffalo; G. E. Helme, Lancaster; L. R. Henderson, Edinburgh; Geo. Herman, Edinburgh; Sidney Hillier, Gloucester; G. J. B. Hope, Edinburgh; J. Q. Hulme, Leek; R. B. Huxtable, Edinburgh: P. R. Jaganonadham, Edinburgh; J. L. Jones, Aberystwyth; H. S. W. Jones, Jersey; A. B. Kenworthy, Southport; Edwd. Kinmont, Edinburgh; L. J. Lamrock, Edinburgh; E. P. T. Von Landsberg, Cape Town; Robt. Lamb New Zealand; H. E. Lee, Worcester; Cyril Lewis, Carmarthen; John Livingstone, Perth: A. J. McAllum, Keith; T. F. Macdonald, Edinburgh; H. M. MacGill, Musselburgh; W. MacGill, Musselburgh, T. N. Macgowan, London; J. T. McKay, Dumfries; T. G. McKellar, Edinburgh; D. J. Mackenzie, Inverness; F. J. McKettrich, Lochmaben; Archd. Mackintosh, Edinburgh; John Maclaren, Edinburgh; Sam. Maclean, Edinburgh: W. B. Macleod, Edinburgh; Chas. Macmaster, Perth; D. Macmillan, New Galloway; J. L. Macrae, Edinburgh; F. R. Mallett, Bolton; Chas. Martin, Birmingham; T. G. Matthews. Westmoreland; J. S. Maynard, Suffolk; Geo. Melville, Edinburgh; Wm. Mill, Dunedin, New Zealand; Jas. Miller, Falkirk; T. H. Milroy, Edinburgh; W. C. Milroy, Dumfriesshire; G. H. Mitchell, Melrose; E. C. Moore, Derby; L. P. More, Kettering; P. St. C. More, Kettering; F. W. Moss, Mussoorie, India; J. D. R. Murro, Nantwich; J. A. Murison, Edinburgh; W. Murray, Montrose; A. J. van Niekerk, Cape Colony; S. J. Oddie, Leeds; W. H. Ogilvie, Edinburgh; John Orr, Edinburgh T. L. Parry, North Wales; W. Peart-Thomas, Gloucester; G H. Prance. Cheltenham; R. L. Price, Oswestry; Harry Rainy, Edinburgh; J. M. Renton, Edinburgh; E. H. Ridley, Bournemouth, J. H. Roberts, Northampton; A. G. Robertson, Edinburgh; J. F. Robertson, Shetland; W. F. Robertson. Drem.; F. A. Rodriguez, Trinidad; J. C. Rossie, Orkney; A. Rutherford Dumfries; Alfred Rutter, Durham; J. B. Scott, Newport, Fife; R. W. L. Scott, Edinburgh; S. A. Shiach, Elgin; E. W. Slayter, Halifax, Canada; J. B. Smith, Kinghorn; J. G. Smith, Dundee; John Spurway, Kidderminster; E. M. Steven, Montrose; Thos. Stodart, Pencaitland, N.B.; A. Stodart-Walker, Edinburgh; Charles Stuart, Perth; D. Stuart, Grantour-on-Spey; C A. Sturrock, Edinburgh; C. D. Sutherland, Aberdeen; John Sutherland, Larbert; W. S. Syme, Kilmalcolm; Bernard Thomas, Llandudno; W. Thyne, Herts; D. D. Tindal, Montrose; H. S. Walker, Spilsby, Lincs; T. D. Walker, Canada; W. J. Kincardine; D. H. Walsh, Earl's Court; G. S. Walton, Salop; G. G. Watson, Edinburgh; N. P. Watt, Edinburgh; A J. Webster, Hornsey Rise; J. H. G. Whiteford, Greenock; David Wield, Ayr; R. S. Wilde, Weston-super-Mare; J. C. Williams, North Wales; J. D. Williams, North Wales; J. W. Williams, Bishopsgate Street; G. Wilson, Edinburgh; J. T. Wilson, Tasmania; T. A. M. Wilson, Dundee; J. M. Wood, Dumfries; D. H. Young. South Australia; J. Y. S Young, Ainslie-place; R. J. E. Young, Ainlie-place; F. S. Zaytoun, Edinburgh.

### Royal Colleges of Physicians and Surgeons, Ireland; Conjoint Scheme.

The following have passed the Third Professional Examination: Acheson, Aiken, W. H. Anderson, W. Black. M Brick, S. Butler, E. L. Cambie, A. S. Cooper. W. J. Corbett, C. F. Cowell, J. J. Cullen, M. Ferguson, A. Gibbs, T. E. Goode, G. Hamilton, W. H. Hornibrook, H. G. Le Fanu, F. C. Lenehan, H. B. Ludlow, J. Marnell, T. Meyler, C. A. Molony, A. B. Moore, P. P. Murray, R. McCombe, A. C. McCutchen, J. M. O'Callaghan, R. H. Oliver, A. W. Power, G. Q. Richardson, T. Smyth, F. O. Stoker, W. Trenwella, J. Trumbull, E. H. Tweedy, F. Warren, G. Whyte, and J. C. Woodside.

### ATHLETICS.

### Cricket.

*Sheffield Medicals v. Leeds Medicals.*—The students from Sheffield played their annual cricket match at Leeds against the students of that town on July 11th. The result was a draw. Score: Sheffield—H. B. Boynton-Lee, b Libbey, 1; G. M. Wilcockson, b Libbey, 1; E. Morton, b Libbey, 19; W. Mettam, b Libbey, 0; F. Edmunds, h.w., b Connell, 8; S. Fox, c Bean, b Fawcett, 29; B. Watts, c Connell, b Smith, 9; G. Cubley, not out. 9; T. P. Stokes, c Smith, b Fawcett, 10; C. Dearden, not out, 5; G. White to bat; extras, 12; total, 103. Leeds—Abbott, b Edmunds, 0; Bean, b Morton, 2; Smith, b Edmunds, 16; Fawcett, b Morton, 0; J. Ellison, b Edmunds, 11; Libbey, not out. 8; Connell, run out, 0; Green, not out, 17; A. Ellison, Phillips, and Jackson, to bat; extras, 3; total, 57.

*Foreign v. Local Students* —Played at the N.D.C.M ground, Gosforth, and resulted in a win for the Locals, of University of Durham College of Medicine. Score: Foreigners—Rolleston (Barts.), b Race, 2; Taylor (Edin. University), c McNabb, b Bryant, 6; Jones (Middlesex), b Race 7; Whitling (Barts.), b Bryant, 5; De Santi (Barts), c Race, b Bryant, 11; J. H. Watts (Owen's College), b Race 11; Badcock (London), b Race, 2; Bishop (Queen's, Birm ) c Lloyd Williams, b Bryant, 5; Cameron (St. Mary's), stumped Lloyd Williams, b Race, 0; Fennings (St. Mary's), c Gibbs, b Bryant, 8; Leigh (University College), not out, 2; extras, 1; total, 60. Locals—Smurthwaite, l b w, b Rolleston, 4; Bryant, c Fennings, b Rolleston, 0; Willis, c Rolleston, b Whitling, 30; Race, b Rolleston, 23; Lloyd Williams, b Whitley, 0; Hedley, c Fennings, b Watts, 9; Mitchell, b Watts, 3; Gibbs, b Rolleston, 7; Visser, b Rolleston, 1; McNabb, c Taylor, b Rolleston, 6; Battiscombe, not out, 10; extras, 2; total, 105.

New York Medical Journal.

A Weekly Review of Medicine.

Vol. LIV., No. 1. Whole No. 657.

SATURDAY, JULY 4, 1891.

Yearly Subscription, Dols. 5.00. Single Copies, Ten Cents.

### THE TREATMENT OF GASTRIC ULCER BY RECTAL ALIMENTATION.

By C. H. ROBINSON, F.R.C.S., M.R.C.P.I.,

DUBLIN, IRELAND.

FELLOW OF THE ROYAL ACADEMY OF MEDICINE IN IRELAND; FELLOW AND EX-EXAMINER, AND REGISTRAR, SCHOOLS OF SURGERY, ROYAL COLLEGE OF SURGEONS IN IRELAND, ETC.

It is a well-recognized law of surgery that a part inflamed should be set at rest, and in the same way where an ulcer of the stomach is present, if food is taken by the mouth the movements of that viscus and the changes caused by digestion must necessarily tend to destroy the cicatricial tissue which may exist. It follows from this that, where circumstances permit, the inflamed and ulcerated part requires rest until such time as a reparative process has been established. This is a common-sense view of the matter, and of late the treatment of gastric ulcer has been successfully carried out by stopping all food by the mouth, and relying on rectal nutritive enemata for supplying the necessary nourishment.

In July of last year a lady, aged twenty-eight, married, with three children, who resided in the County Wexford, came under my care with the principal symptoms of gastric ulcer, and whose illness had lasted about four years. She had been treated by several physicians, but showed no improvement, and her case was regarded by all whom she consulted as one of ulceration of the stomach. She suffered from vomiting, which mostly occurred night and morning; after eating she was obliged to lie down to try and prevent the contents of the stomach being ejected. The pain, which was localised to one particular part of the epigastric region, came on in paroxysms, was increased by pressure, by food, by exercise, and by mental anxiety. When brought on by food it lasted about an hour and a half, vomiting giving relief, while it was lessened by lying down. On making a careful examination, no tumour could be detected.

I prescribed a mixture containing pepsin, bismuth, and hydrocyanic acid with morphine, food to be taken in very small quantities, and such as would be easily digested. In a few days she returned to her home, and I did not see her until the 30th of September, when I found that, although somewhat improved, she still suffered a good deal. Some cases of gastric ulcer published in the *Lancet* at this time interested me considerably, and I determined to use the same method of treatment—viz., by rectal nutritive enemata, withholding all food from the mouth. I explained the *rationale* of this method of treatment to my patient, and she agreed to be guided by me.

On the 4th of October, her weight being eight stone ten pounds the previous day, she commenced using enemata of "Caffyn's liquor carnis" and milk every fourth hour, every second day using once a dessertspoonful of brandy in a wineglassful of milk instead of the usual dose of liquor carnis, all food by the mouth being strictly forbidden. On the evening of the 9th of October the pain, which had been absent ever since she commenced this treatment, returned for a short time, which she attributed to having sucked a lozenge, but it did not again recur, and on the 17th of October, the fourteenth day of the treatment, she was permitted to take a little food by the mouth, meat being interdicted for a fortnight, the enemata of the liquor carnis and milk to be used twice daily for five days longer.

Two days afterward she returned to her home in Wexford, seventy miles distant, and on the 28th of October I received a letter from her in which she said: "My stomach is quite well. I have not felt pain since I came home . . . My appetite is increasing. I still continue to take the liquor carnis."

During the treatment she did not keep her bed, and, although rather weak, did not appear to suffer from the withdrawal from food by the mouth.

There was no hæmorrhage, and therefore some might be disposed to question whether this was a case of gastric ulcer at all; but I think, on reviewing the history, viz., the duration of the malady, the localized pain increased by pressure and by food, the failure of all medicines to remove the various symptoms—that few will disagree with my diagnosis of the case.

Dr. Donkin, physician to the Westminster Hospital, London, who has had great experience in cases of the kind, says that it is always wise to recognize in every protracted case of localized and unerrant pain after taking food, continuing for any length of time, and especially when followed and relieved by vomiting, a possible gastric ulcer. Vomiting of blood, although a very important sign, as a matter of safe practice must not be looked upon as a necessary for the diagnosis of the disease.

In conclusion, I may add that my patient is now in excellent health, and her weight is nine stone six pounds, or an increase of ten pounds in about six months.—*Reprinted from The New York Medical Journal.*

